# Controlled Coffee Intake Enhances Erythrocyte Deformability, Na,K-ATPase Activity, and GSH/GSSG Ratio in Healthy Young Adults

**DOI:** 10.3390/biomedicines12112570

**Published:** 2024-11-09

**Authors:** Dominika Radosinska, Tomas Jasenovec, Alzbeta Golianova, Ivan Szadvari, Rastislav Vazan, Ivona Kovacicova, Denisa Snurikova, Norbert Vrbjar, Jana Radosinska

**Affiliations:** 1 Institute of Medical Biology, Genetics and Clinical Genetics, Faculty of Medicine, Comenius University in Bratislava, Sasinkova 4, 811 08 Bratislava, Slovakia; dominika.radosinska@fmed.uniba.sk; 2 Institute of Physiology, Faculty of Medicine, Comenius University in Bratislava, Sasinkova 2, 811 08 Bratislava, Slovakia; tomas.jasenovec@fmed.uniba.sk (T.J.); golianova28@uniba.sk (A.G.); ivan.szadvari@fmed.uniba.sk (I.S.); rastislav.vazan@fmed.uniba.sk (R.V.); 3 Centre of Experimental Medicine, Slovak Academy of Sciences, Dúbravská Cesta 9, 841 04 Bratislava, Slovakiadenisa.snurikova@savba.sk (D.S.); norbert.vrbjar@savba.sk (N.V.)

**Keywords:** erythrocytes, coffee, sodium–potassium pump, deformability, nitric oxide, oxidative stress, GSH/GSSG

## Abstract

Background: Published studies suggest that regular coffee consumption may reduce the risk of various diseases. However, many of these studies relied on questionnaire-based data, limiting their ability to identify the specific biological mechanisms behind the observed effects. This study focuses on controlled coffee consumption among healthy young adults to clarify its effects on erythrocyte properties. The functional condition of erythrocytes is important as it affects both macro- and microcirculation. Additionally, since erythrocytes are not true cells, they are particularly sensitive to biochemical and biophysical changes when exposed to biologically active substances. Methods: After a washout period, 33 healthy young volunteers were asked to consume a standardized dose of a coffee beverage daily for 3 weeks. Basic hematological and body composition parameters were recorded before and after the intervention. Erythrocyte functional status was evaluated based on the following measurements: deformability, osmotic resistance, Na,K-ATPase activity, and nitric oxide production, along with monitoring oxidative stress markers. Results: After a coffee consumption period, both erythrocyte count and hematocrit value increased, while body composition remained unchanged. Erythrocyte deformability improved across a range of shear stress values typical of human circulation. This improvement was accompanied with enhanced Na,K-ATPase activity in erythrocyte membranes in the wide range of sodium ion concentrations, as well as increased nitric oxide production by erythrocytes. Additionally, a higher GSH/GSSG ratio, indicating a shift towards a more favorable antioxidant balance, was observed in erythrocytes following the coffee intake period. Conclusions: The results of this study suggest that controlled coffee intake in healthy young adults can positively influence various indices of erythrocyte functional status. Although the observed statistically significant changes were modest, the findings consistently indicate a positive modulation of erythrocyte properties—cell deformability, oxidative resilience, and active membrane transport of cations—following coffee consumption.

## 1. Introduction

It is well known that lifestyle plays a significant role in influencing various diseases, with nutrition being a key component of daily life. For many people, coffee is an integral part of their daily routine and ranks among the most widely consumed beverages worldwide [1]. Beyond caffeine, coffee contains a diverse array of compounds [2], some of which provide health benefits, such as chlorogenic acids, polyphenols, diterpenes, micronutrients, melanoidins, and fiber. However, there are also components, such as lipids in unfiltered coffee and acrylamide produced during the roasting process, that may have less favorable effects [3].

Published studies suggested that regular coffee drinking may reduce the risk of multiple diseases, including Parkinson’s disease, type 2 diabetes mellitus, chronic liver diseases, and cardiovascular diseases [4,5,6,7]. Additionally, coffee intake has been associated with lower all-cause mortality, particularly reducing the risk of death from digestive and circulatory diseases [8]. Revealing potential mechanisms responsible for such effects could represent a problem, as many studies relied on questionnaire-based data collection regarding coffee intake. Such studies are limited in their ability to identify the specific biological mechanisms underlying the observed effects attributed to coffee intake and its impact on health outcomes. This study is based on controlled coffee drinking in healthy young adults and aims to explain its effects on the properties of erythrocytes. The rationale for focusing on erythrocytes lies in the fact that, following coffee ingestion and subsequent absorption of its constituents into blood, erythrocytes are directly exposed to all absorbed biologically active compounds from coffee. Moreover, unlike other nucleated cells, erythrocytes lack repair mechanisms, making them sensitive to biochemical and biophysical changes when exposed to potentially biologically active substances. Due to their short lifespan, erythrocytes can be useful and sensitive indicators of overall health. Furthermore, the functional status of erythrocytes was repeatedly shown to be highly related to the quality of microcirculation [9]. This study examines a range of erythrocyte parameters to provide a comprehensive and in-depth understanding of potential mechanisms that may be influenced by coffee components. Beyond basic parameters, such as erythrocyte count and volume, hematocrit value, and variations in cell size and volume, particular attention was given to erythrocyte deformability, which reflects the cells’ capacity to change shape under external forces, thereby affecting blood flow throughout the circulatory system. Well-deformable erythrocytes help reduce blood viscosity [10], which contributes to maintaining steady blood flow in larger vessels. In microcirculation, erythrocyte deformability directly affects erythrocyte passage through the small capillaries.

Among the factors that interfere with or contribute to proper erythrocyte deformability, the balance of cation concentrations across the plasma membrane, maintained by the activity of the sodium–potassium pump (Na,K-ATPase), has been shown to be significant [11,12], alongside nitric oxide (NO) bioavailability [13]. Research suggests that caffeine may modulate Na,K-ATPase enzyme differently across various body regions, with inhibition observed in the brain [14], but stimulation reported in the kidney [15] and skeletal muscle [16]. Therefore, investigating the effect of coffee drinking on erythrocyte Na,K-ATPase appears to be valuable for further understanding its potential impacts. In addition to its well-known effects on vascular smooth muscle, NO is thought to influence circulation via the regulation of erythrocyte deformability [17]. Coffee intake appears to influence NO turnover in the body, as exhaled NO levels were significantly reduced within the first hour after consumption. This effect is attributed to caffeine, as decaffeinated coffee showed no impact on exhaled NO levels [18]. Erythrocyte properties are also sensitive to shifts in the balance between pro-oxidants and antioxidants [12]. Caffeine, along with other components in coffee beans, has been shown to be a potent antioxidant [19], potentially modulating erythrocyte properties.

Considering the known facts, this study aims to investigate potential changes in erythrocyte properties, specifically examining their deformability, Na,K-ATPase activity, and NO production, while monitoring oxidative stress markers following a 3-week period of controlled coffee intake in healthy young volunteers.

## 2. Materials and Methods

### 2.1. Study Design

The study included 33 healthy young adults with a median age of 22 years (IQR: 21; 23), comprising 8 males and 25 females. Participants were included in the study for the following eligibility criteria: university students, no obesity (BMI under 30 kg/m^2^), absence of chronic and acute illnesses (hypertension, diabetes, coronary heart disease, cerebrovascular disease, or other major diseases), no pharmacologic treatment (any drugs or supplements with laxatives, anti-inflammatory and lipid-reducing properties, antibiotics), and no vitamin supplements. Participants were asked not to change their dietary habits and physical activities during the study, with adherence to this guideline being regularly monitored.

Two weeks before the experiment, participants were instructed to gradually reduce their intake of coffee and polyphenol-rich foods and to completely abstain from both one week before the experiment. At the beginning of the experiment, venous blood samples were collected in the morning after a 12-hour fast, using tubes containing ethylenediaminetetraacetic acid as an anticoagulant (Sarstedt, Nümbrecht, Germany).

During the experiment, participants consumed a standardized coffee brew twice a day: one in the morning, before or with the first meal, and one after lunch, without a strictly fixed schedule. At the end of the experiment, venous blood samples were collected again for post-intervention analyses. Additionally, body composition was assessed using bioelectrical impedance analysis, performed with the InBody Scale (InBody Co., Ltd., Seoul, South Korea), both before and after the standardized coffee consumption period. The same dataset was compared pre- and post-intervention to evaluate changes within the same individual. The experiments were not randomized, and investigators were only partially blinded to the sample allocation during experiments. The experiments did not include a non-intervention group.

Whole blood was used to determine basic erythrocyte parameters on Sysmex F-820 (Sysmex Corp, Tokyo, Japan), as well as to assess erythrocyte deformability and NO production. The remaining blood was centrifuged (1150× *g*, 5 min, 4 °C). The erythrocytes were washed three times in a 0.9% NaCl solution and then provided for the assessment of osmotic resistance, the preparation of 10% hemolysates in cold distilled water for the determination of oxidative stress markers, and the isolation of erythrocyte membranes for measuring Na,K-ATPase kinetic parameters.

The study was approved by the Ethics Committee of Faculty of Medicine, Comenius University and University Hospital in Bratislava, Slovak Republic (protocol code: 25/2023, date of approval: 2 February 2023). Informed consent was obtained from all individuals. All procedures were performed in accordance with the Declaration of Helsinki.

### 2.2. Coffee Brew Preparation

A commercially available coffee blend (70% arabica Brasilia, 30% arabica Honduras) was used to prepare the coffee brew. The coffee beans were medium roasted (200–201 °C, 11 min), ground, and distributed to participants, who received a fresh portion of ground coffee twice a week. Each participant was given instructions on how to prepare the brew and completed a supervised session to ensure consistency in the brewing method. All participants used the same household coffee maker to filter coffee, with 12 g of coffee per 120 mL of water per serving.

### 2.3. Osmotic Resistance of Erythrocytes

Osmotic resistance was assessed by exposing washed erythrocytes to NaCl solutions ranging from 0% to 0.9% for 30 min at room temperature. Following centrifugation, the degree of hemolysis in the supernatants was measured spectrophotometrically at 540 nm. The supernatant from the 0.9% NaCl solution was used as a baseline for no hemolysis, while distilled water (0% NaCl) was used as the reference standard for 100% hemolysis. IC_50_ values, representing the NaCl concentration at which 50% hemolysis occurred, were calculated from the obtained data as previously reported [20].

### 2.4. Measurements of Na,K-ATPase Kinetics in Erythrocyte Membranes

Isolation of erythrocyte membranes was performed in a series of hypotonic solutions as described previously [20]. The activity of Na,K-ATPase in erythrocyte membranes was evaluated in the presence of increasing concentrations of NaCl (2.0–100.0 mmol/L) as a cofactor. Samples containing 50 μg/mL of membrane protein were diluted in an incubation buffer and pre-incubated for 20 min at 37 °C without ATP. Following 20 min of incubation with ATP (in final concentration 8 mmol/L), the reaction was halted by adding a 12% trichloroacetic acid. The released inorganic phosphorus resulting from ATP hydrolysis was spectrophotometrically measured at a wavelength of 700 nm, as described previously [20].

The obtained data on Na,K-ATPase activity at a whole range of Na^+^ concentration were used to generate kinetic curves and to derive the kinetic parameters of the Na,K-ATPase enzyme based on the Michaelis–Menten equation: V_max_ (the maximum reaction velocity of the enzyme) and K_Na_ (the Na^+^ concentration required for half-maximal enzyme activation). V_max_ indicates the number of active Na, K-ATPase molecules, while K_Na_ reflects the enzyme’s affinity for its cofactor.

### 2.5. Erythrocyte Deformability

Erythrocyte deformability was assessed using a Laser-Optical Rotational Red Cell Analyzer (Lorrca MaxSis, RR Mechatronics, Zwaag, The Netherlands). The method is based on rotating the erythrocytes between two concentric cylinders in a medium of known viscosity while applying an exact shear stress. Under stress, erythrocytes deform, transitioning from their native biconcave disk shape to an elongated form. A laser beam generates a measurable diffraction pattern, from which the Elongation Index (EI) is calculated according to the length and width of fitted elongated erythrocytes: EI = (length − width)/(length + width). This study examined the EI at three levels of shear stress: 0.3, 3, and 9.49 Pa.

### 2.6. Fluorescence Analysis of NO Production

A 10 µL portion of whole blood was used to analyze NO production by erythrocytes, utilizing the fluorescent probe DAF-2 DA (25 μmol/L, Abcam, Cambridge, UK), as previously described [20].

### 2.7. Oxidative Stress Markers in Erythrocytes

A series of specific oxidative stress markers was examined to assess the intervention’s impact on various aspects of redox signaling in erythrocytes. Thiobarbituric acid-reactive substances (TBARS) were used as a marker of lipid oxidative damage, being by-products of lipid peroxidation. For protein oxidative damage, advanced protein oxidation products (AOPP) were measured. The ferric reducing antioxidant power (FRAP) assay served as a general indicator of antioxidant status, assessing the sample’s ability to reduce ferric ions (Fe^3^^+^) to ferrous ions (Fe^2^^+^). The ratio of reduced to oxidized glutathione (GSH/GSSG ratio) was used to evaluate antioxidant levels, focusing on the sulfhydryl/disulfide balance within erythrocytes.

Washed erythrocytes were hemolyzed in cold distilled water to obtain a 10% hemolysate. The hemolysates were subsequently diluted for the following measurements: TBARS were diluted 100-fold, AOPP 400-fold, and GSH/GSSG and FRAP 200-fold. All parameters were determined according to previously established methods [20].

Protein oxidation (AOPP) was measured spectrophotometrically at 340 nm. Samples were diluted 1:4 in phosphate-buffered saline, mixed with glacial acetic acid, and calibrated using Chloramine T (0–100 µmol/L) with potassium iodide.

Lipid peroxidation, measured via TBARS, was determined using fluorescence at ex = 515 nm and em = 553 nm. Samples were mixed with deionized water, 0.67% thiobarbituric acid, and glacial acetic acid, vortexed, incubated at 95 °C for 45 min, and then cooled. After adding n-butanol, samples were centrifuged at 4 °C (2000× *g* for 10 min), and the organic phase was measured. A 1,1,3,3-tetraethoxypropane (0–10 µmol/L) standard was used.

FRAP was measured as a general indicator of erythrocyte antioxidant capacity. Fresh FRAP reagent (3 mol/L acetate buffer, 10 mmol/L 2,4,6-Tris(2-pyridyl)-s-triazine solution, 20 mmol/L ferric chloride hexahydrate) was warmed to 37 °C, and absorbance was measured at 593 nm as blank. Standards (0–1000 µmol/L FeSO_4_·7H_2_O) and samples were added, and mixed, and after 4 min, absorbance was re-measured at 593 nm.

The GSH/GSSG ratio was used to assess antioxidant status. For GSH, samples were mixed with 1 mg/mL O-phthalaldehyde and 100 mmol/L phosphate-buffered saline containing 2.5 mmol/L EDTA-Na_2_. After a 15-minute incubation in the dark at room temperature, fluorescence was measured at ex = 350 nm and em = 460 nm. For GSSG, samples were mixed with 5 mg/mL N-ethylmaleimide, incubated for 40 min in the dark at room temperature, and transferred to a dark microplate with 1 mg/mL O-phthalaldehyde and 0.1 M NaOH. After 15 min of incubation, fluorescence was measured at ex = 350 nm and em = 460 nm.

### 2.8. Statistical Analyses

The sample size was not calculated prior to the experiments. However, a retrospective power analysis was conducted using the sample size calculator for paired data [21] to assess whether the sample size is adequate for drawing statistically reliable conclusions. A sample size of 21 pairs was determined to be sufficient to achieve a power of 80% and a two-sided significance level of 5% for detecting an effect size of 0.66 and higher between pairs, unless stated otherwise.

Data are presented as the mean ± standard deviations, or as medians with interquartile ranges (Q1; Q3), as appropriate. Normality was assessed using the Shapiro–Wilk test, and outliers were identified with the Grubbs test. Group differences were analyzed using either the paired *t*-test or the Wilcoxon test, depending on data normality. Differences were considered statistically significant at *p* < 0.05.

## 3. Results

### Characteristics of Study Participants

In body composition parameters, no difference was noted before and after the experiment—see Supplemental Table S1.

**Figure 1:**
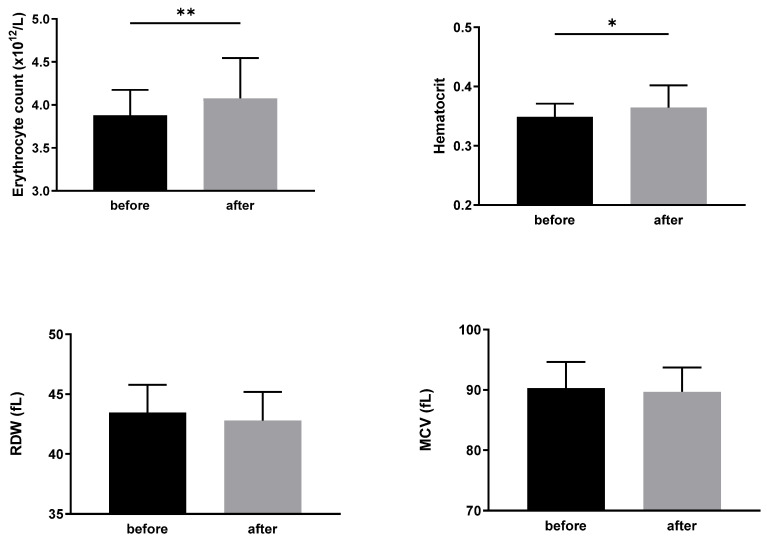
The count of erythrocytes, hematocrit value, red cell distribution width (RDW) and mean cell volume (MCV) in study participants before and after the standardized coffee intake. Data are presented as the means ± standard deviations. Statistical significance: * *p* < 0.05, ** *p* < 0.01.

Regarding the basic hematological parameters related to erythrocytes, their count, hematocrit value, red cell distribution width (RDW), and mean cell volume (MCV) were determined. The count of erythrocytes and hematocrit value were higher after the coffee drinking, while RDW and MCV remained unchanged—see Figure 1.

The ability of erythrocytes to resist the changes in osmotic pressure—osmotic resistance, was not modified after the period of coffee intake. The value IC_50_ at the beginning of the experiment was 0.462 ± 0.01% of NaCl, while it remained almost the same—0.461 ± 0.01% of NaCl, at the end of the experiment (*p* = 0.5).

**Figure 2:**
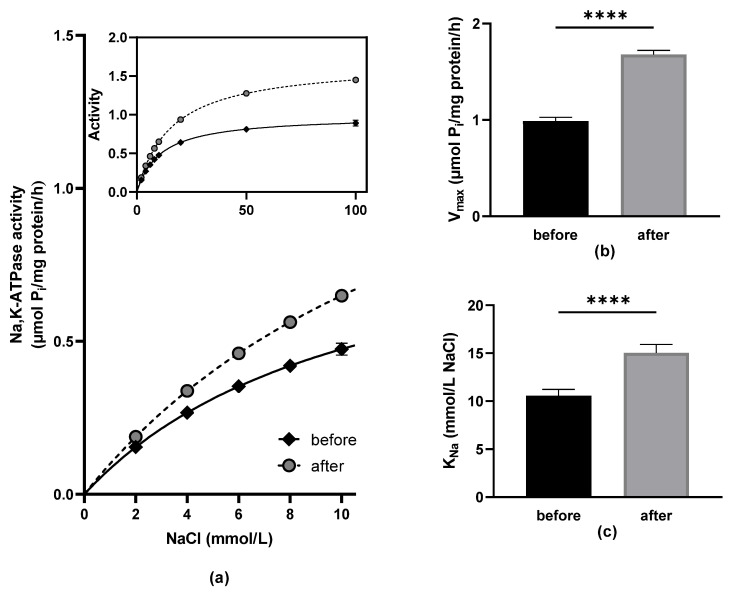
The Na,K-ATPase enzyme kinetics in study participants before and after the standardized coffee intake. (**a**) Activation of Na,K-ATPase enzyme in Na^+^ concentrations ranging from 2 to 10 mmol/L. Insert: an activation of the enzyme in the whole investigated NaCl concentration range. (**b**) Kinetic parameters of erythrocyte Na,K-ATPase enzyme V_max_—maximal reaction velocity and (**c**) K_Na_—NaCl concentration required for half-maximal activation of Na,K-ATPase. Data are presented as mean ± standard deviations. Statistical significance: **** *p* < 0.0001.

Na,K-ATPase activity was higher in samples collected after three weeks of controlled coffee consumption compared to those collected from the same subjects following a two-week washout period. This increase was observed across the full range of sodium ion concentrations when the enzyme was activated by rising NaCl levels (Figure 2a). At the lowest NaCl concentration (2 mmol/L), the stimulation represented 22% (Na,K-ATPase activity in μmol of inorganic phosphate/mg protein/h: 0.15 before vs. 0.19 after the coffee consumption). The stimulatory effect gradually grew with increasing the concentration of NaCl reaching the level of 63% observed at the highest concentration of NaCl—100 mmol/L (0.89 before vs. 1.45 after the coffee consumption) (Figure 2a—insert). Comparing both kinetic parameters, V_max_ and K_Na_ values, we observed that the V_max_ was higher by 70% and the K_Na_ increased by 47% in samples collected after the controlled coffee drinking (Figure 2b,c).

**Figure 3:**
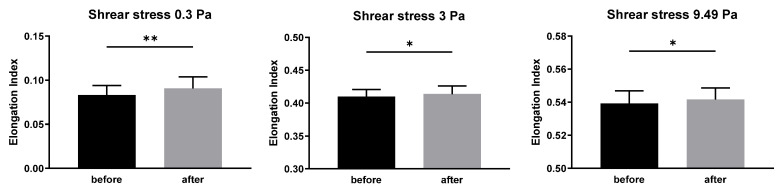
Elongation index of erythrocytes at different levels of shear stress—0.3, 3, and 9.49 Pa, in study participants before and after the standardized coffee intake. Data are presented as the means ± standard deviations. Statistical significance: * *p* < 0.05, ** *p* < 0.01.

Regarding the viscoelastic properties of erythrocytes, the elongation index at all levels of shear stress was higher after the period of coffee intake in comparison with the corresponding values at the beginning of the experiment (Figure 3).

Quantitative analysis of the DAF-2 DA fluorescent signal indicated that NO production by erythrocytes was more intense at the end of the experiment compared with the levels observed before the coffee intake (in arbitrary units: 54.76 (53.69; 56.97) before, versus 54.98 (54.28; 57.41) after the coffee intake, *p* = 0.03).

The lipid peroxidation parameter, measured as TBARS, and the protein oxidation parameter, AOPP, did not show any significant differences before and after the intervention. Similarly, the marker for ferric-reducing antioxidant power (FRAP) remained unchanged before and after coffee consumption. However, oxidized glutathione levels were significantly lower following coffee intake, while the ratio of reduced and oxidized glutathione—a thiol–disulfide balance (a general marker of the cell’s redox environment)—was significantly elevated after the intervention (Table 1).

## 4. Discussion

Coffee is one of the most widely consumed beverages worldwide, renowned for its neurostimulant properties. Rich in various biologically active compounds, coffee has been linked to both positive and negative effects on cells within the organism, though these impacts remain a topic of debate. For instance, chlorogenic acid, a compound with significant antioxidant properties, appears to be a double-edged sword. On one hand, it has been shown to offer protective effects on erythrocytes [22], while on the other, it may cause DNA damage and induce carcinogenic effects [23]. Another key component of coffee is caffeine (1,3,7-trimethylxanthine), a purine alkaloid with well documented pharmacological effects, including cytoprotective and antioxidant properties. A previous study investigating the influence of medium-light and medium-roast paper-filtered coffee on antioxidant capacity and lipid peroxidation in healthy volunteers demonstrated antioxidant benefits from both types of coffee [24].

This study aimed to deepen the understanding of coffee’s effects by exploring newly selected characteristics of erythrocytes in young healthy volunteers. The decision to use the filtration method for coffee preparation was based on previously published evidence regarding its composition and health benefits, as well as the need for uniformity to ensure that the coffee brew was prepared consistently for all study participants. To maintain this consistency, all participants used the same simple-to-operate coffee maker, which required minimal supervision. This approach helped standardize the brewing process. The filtration method also offered additional health benefits, as unfiltered coffee is known to contain higher concentrations of diterpenes, compounds that, despite their beneficial pharmacological properties, are associated with increased levels of plasma triglycerides, total cholesterol, and LDL cholesterol—generally considered undesirable effects [25,26]. Additionally, filtered coffee has a lower ash content, contributing to a more appealing taste for the average consumer.

Nutrition and lifestyle significantly impact the functional condition of erythrocytes [13,27,28]. With this in mind, strict adherence to instructions was set as one of the inclusion criteria. To ensure consistency, participants were instructed to maintain their usual lifestyle habits, including dietary patterns, from the start of the wash-out period until the end of the experiments. Additionally, participants were asked to report on their lifestyle choices throughout the study, providing details on diet, physical activity, and sleep patterns. This design allowed for natural lifestyle variations among participants, effectively serving as controls within a ‘before-and-after’ observation framework. By incorporating these real-life complexities, rather than relying solely on controlled laboratory conditions or strict dietary restrictions, this approach enabled us to draw more meaningful conclusions about the effects of coffee consumption as an intervention. Regarding the duration of the experiment, studies with a 3-week or shorter period of coffee consumption showed its positive effect on glucose metabolism and redox balance [29], prevention of DNA damage by oxidation [30,31], or increased plasma antioxidant capacity [32] in healthy participants. Thus, this 3-week time interval was deemed sufficiently long for the purposes of this study.

The body composition parameters were registered to assess the possible effects of coffee intake on body mass index, body fat, skeletal muscle mass, and fluid balance. However, unchanged parameters suggest no significant impact on body composition after 3 weeks of coffee intake in healthy young adults.

The determination of basic erythrocyte parameters revealed an unexpected increase in both erythrocyte count and hematocrit value following the period of controlled coffee consumption. An exploration of the mechanisms underlying this finding suggested multiple potential explanations. Caffeine was shown to inhibit eryptosis (the suicidal death of erythrocytes) following glucose depletion [33]. Additionally, a beneficial correlation between antioxidant capacity and caffeine levels has been observed in stored erythrocytes [34]. As an antagonist of adenosine receptors, caffeine can interfere with adenosine signaling, which has been shown to inhibit erythropoiesis (the formation of erythrocytes) [35]. Therefore, caffeine intake may counteract this inhibition, ultimately leading to the increased erythrocyte count observed in this study.

Regarding the measurement of erythrocyte deformability, this study focused on the ability of erythrocytes to change their shape under three degrees of shear stress. Under physiological conditions, shear stress in humans can vary significantly, ranging from 0.1 to 9.5 Pa [36]. Shear stress is defined as the force applied parallel to a material’s cross-section, resulting naturally from blood flow in circulation. In laminar blood flow, shear stress is also defined as the force exerted by one layer of fluid against an adjacent layer. Ektacytometry is a method that enables the observation of changes in the deformability of erythrocytes across a selected range of shear stress values. This study focused on three degrees of applied force against erythrocytes: the lowest value of 0.3 Pa was referred to correspond to an average value of shear stress in large veins, while its higher value of 3 Pa is typical for compliant vessels with smaller diameters, such as small venules. The highest value, 9.49 Pa, corresponds to the maximal shear stress observed in small resistant vessels like arterioles, as well as the smallest capillaries [37]. It should be noted that to detect statistical differences at higher shear stress values in this study, a larger number of participants would be needed to achieve the desired 80% power. However, the consistent trend of statistically significant before-and-after observations suggests an improvement in erythrocyte deformability across the range of shear stress. Furthermore, although the study participants were healthy young adults with no history of disease—leaving limited possibilities for significant improvement following any intervention—the observed increase in erythrocyte deformability (improvement in the viscoelastic properties of erythrocytes) across the shear stress range is an intriguing finding. One might speculate that the effect of coffee consumption on erythrocyte deformability could be even more pronounced in individuals with initially impaired deformability.

Looking for the mechanisms that could be responsible for the enhanced property of erythrocytes to change their shape, this study focused on the functionality of Na,K-ATPase enzyme. This enzyme, being responsible for the active transport of sodium and potassium cations across the erythrocyte membrane, plays a significant role in the maintenance of the normal volume of erythrocytes as well as the cytoplasmic viscosity—both determinants of erythrocyte deformability. Considering the influence of controlled three-week lasting coffee consumption on functional properties of the Na,K-ATPase in erythrocytes of healthy volunteers, our data indicate that the increased enzyme activity may be a consequence of elevation in the number of active Na,K-ATPase molecules in erythrocytes, as suggested by significantly higher V_max_ values. However, the affinity of the Na^+^-binding site seems to be lower after the consumption of coffee, as shown by significantly increased K_Na_ values. Thus, the enzyme in erythrocytes subjected to coffee drinking seems to be naturally adapted to the possible higher intracellular concentration of Na^+^ because the Na,K-ATPase can increase its activity in the range where the enzyme in erythrocytes collected after the washout period is already saturated with Na^+^.

Due to the high ability of erythrocytes to sensitively react to changes in a balance of pro-oxidants and antioxidants, as well as due to the well-known ability of coffee constituents to modify the redox balance, this study also focused on changes in basic oxidative stress markers within the erythrocytes. GSH is the most abundant endogenous thiol, and one of the key intracellular antioxidants. In redox reactions, monomeric GSH is oxidized to form glutathione disulfide—GSSG [38]. The significant increase in the GSH/GSSG ratio after the period of coffee intake is in line with a previously observed positive correlation between caffeine and glutathione levels [34]. Another study showed that cafestol and kahweol that are found in coffee beans increase glutathione transferase, as well as c-glutamylcysteine synthetase activity, which was shown to result in elevated GSH levels [39]. Another randomized, longitudinally designed human intervention trial also confirmed an increase in total glutathione level—especially after the intake of dark roast coffee beverage [40]. The consumption of dark roasted, naturally low-caffeine coffee resulted in a greater GSH/GSSG ratio in erythrocytes, a change not observed with the same type of light roasted coffee [41]. This suggests that it is not only caffeine but also the substances generated during the roasting process, such as melanoidin and N-methylpyridinium, that are likely responsible for this effect. It is worth noting that the increase in the GSH/GSSG ratio was primarily due to a significantly reduced pool of GSSG in erythrocytes, as an increase in GSH content did not reach the level of statistical significance in this study. Markers of lipid peroxidation were not modified after the coffee intake in erythrocytes in this study, as well as in blood plasma in the previously published paper [24].

## 5. Conclusions

The results of this study suggest that controlled coffee intake in healthy young adults can positively influence various functional indices of erythrocytes. Although the observed statistically significant changes were modest and a direct causal link between coffee intake and enhanced erythrocyte function remains unconfirmed, the results reveal a consistent trend of favorable effects. These benefits, observed after a 3-week period of controlled filtered coffee consumption, include improvements in cell deformability, oxidative resilience, and active membrane transport of cations—factors crucial for erythrocyte health. This study presents novel insights into the potential role of coffee as a modifiable dietary component with positive implications for erythrocyte functionality in young adults.

## Figures and Tables

**Table 1:** Parameters of oxidative stress and antioxidant status in erythrocytes in study participants before and after the standardized coffee intake.

Parameter	Before	After	*p*-Value
TBARS (µmol/L)	96.47 ± 13.52	97.23 ± 8.23	0.7360
AOPP (µmol/g)	0.150 ± 0.015	0.149 ± 0.014	0.5269
FRAP (mmol/L)	45.4 (40.8; 47.9)	42.7 (39.2; 44.6)	0.2576
GSH (µmol/L)	6.250 ± 0.617	6.478 ± 0.335	0.0691
GSSG (µmol/L)	11.10 ± 1.52	10.04 ± 1.44	**0.0046**
GSH/GSSG ratio	0.557 ± 0.066	0.644 ± 0.069	**<0.0001**

Abbreviations: TBARS—thiobarbituric acid reactive substances, AOPP—advanced oxidation protein products, FRAP—ferric reducing antioxidant power, GSH/GSSG ratio—the ratio of reduced to oxidized glutathione. Data are presented as means ± standard deviations or medians and interquartile range where appropriate. *p*-values in bold indicate statistically significant differences.

## Data Availability

The data supporting the findings of this study are available in this article.

## Supplementary Materials

The following supporting information can be downloaded at https://www.mdpi.com/article/10.3390/biomedicines12112570/s1. Table S1: Body composition parameters in study participants before and after the 3-week lasting coffee intake.
