# Supplementary material for: Controlled Coffee Intake Enhances Erythrocyte Deformability, Na,K-ATPase Activity, and GSH/GSSG Ratio in Healthy Young Adults
Source: Biomedicines. 2024 Nov 9;12(11):2570. doi: 10.3390/biomedicines12112570 (PMC11591573; doi:10.3390/biomedicines12112570)
Supplement: Supplementary file 1 [file biomedicines-12-02570-s001.zip › biomedicines-3287867-supplementary.pdf]

Supplemental Table S1.

Body composition parameters in study participants before and after the 3-week lasting coffee intake.

**Parameter**	**Before**	**After**	p-value
Body Mass Index	22.6 (20.35; 25.78)	22.4 (20.4; 25.93)	0.18
Total Body Water	33.35 (31.25; 37.05)	32.9 (30.5; 37.15)	0.53
Minerals	3.275 (3.103; 3.503)	3.275 (3; 3.503)	0.07
Body Fat Mass	16.25 (12.6; 25.45)	16.65 (12.43; 26.23)	0.81
Soft Lean Mass	42.9 (40.13; 47.5)	42.3 (38.95; 47.78)	0.58
Fat Free Mass	45.65 (42.65; 50.5)	45 (41.6; 50.63)	0.53
Skeletal Muscle Mass	24.95 (23.1; 27.88)	24.75 (22.33; 28.18)	0.87
Percent Body Fat	27.7 (19.9; 36.33)	27.75 (19.68; 36.28)	0.78

Data are presented as medians with interquartile ranges (Q1; Q3).
